# Predictability of Seasonal Mood Fluctuations Based on Self-Report Questionnaires and EEG Biomarkers in a Non-clinical Sample

**DOI:** 10.3389/fpsyt.2022.870079

**Published:** 2022-04-08

**Authors:** Yvonne Höller, Maeva Marlene Urbschat, Gísli Kort Kristófersson, Ragnar Pétur Ólafsson

**Affiliations:** ^1^Faculty of Psychology, University of Akureyri, Akureyri, Iceland; ^2^School of Health Sciences, University of Akureyri, Akureyri, Iceland; ^3^Faculty of Psychology, University of Iceland, Reykjavík, Iceland

**Keywords:** seasonal mood fluctuations, EEG biomarkers, cognitive vulnerabilities, prediction, machine learning, seasonal affective disorder winter depression

## Abstract

Induced by decreasing light, people affected by seasonal mood fluctuations may suffer from low energy, have low interest in activities, experience changes in weight, insomnia, difficulties in concentration, depression, and suicidal thoughts. Few studies have been conducted in search for biological predictors of seasonal mood fluctuations in the brain, such as EEG oscillations. A sample of 64 participants was examined with questionnaires and electroencephalography in summer. In winter, a follow-up survey was recorded and participants were grouped into those with at least mild (*N* = 18) and at least moderate (*N* = 11) mood decline and those without self-reported depressive symptoms both in summer and in winter (*N* = 46). A support vector machine was trained to predict mood decline by either EEG biomarkers alone, questionnaire data from baseline alone, or a combination of the two. Leave-one-out-cross validation with lasso regularization was used with logistic regression to fit a model. The accuracy for classification for at least mild/moderate mood decline was 77/82% for questionnaire data, 72/82% for EEG alone, and 81/86% for EEG combined with questionnaire data. Self-report data was more conclusive than EEG biomarkers recorded in summer for prediction of worsening of depressive symptoms in winter but it is advantageous to combine EEG with psychological assessment to boost predictive performance.

## 1. Introduction

Winter depression is the most common form of seasonal affective disorder (SAD), characterized by depressive symptoms in winter and remission in spring (1, 2). As compared to major depressive disorder, patients with SAD exhibit atypical depression symptoms, especially hyperphagia and hypersomnia, but scoring lower in interpersonal sensitivity and rejection avoidance (3). The condition has been reported in many regions of the world, with 1–3% of adults being affected in temperate climates (4), and being highly relevant in nordic countries with prevalence rates over 12%, e.g., in Alaska, Denmark, Norway, and Siberia (5–8). The disorder was reported to be occurring over many years for most patients, with full remission within about 9 years being found in 14% of cases, only (9), although a later study suggests higher remission rates (10). Several reports criticize the defined borders between SAD, major depression, and the DSM criteria (11, 12). However, SAD is usually not as severe as major depression but still has socioeconomic implications as it negatively impacts quality of life and was suggested to increase the probability of unemployment (13). Because of the relatively short period of SAD compared to the typical duration of psychotherapy and the long time it takes for serotonine-selective reuptake inhibitors to show an effect it might be wise to start prevention at least two months before onset of symptoms. In turn, this requires early identification of people at-risk to develop SAD. Therefore, the search for characteristics and biomarkers with a high predictive value as well as a better understanding of vulnerabilities for SAD is highly warranted. If we could identify cognitive vulnerabilities for SAD, specific designs for psychotherapy could be developed. Both, the early estimation of the risk for SAD, and suggestions toward an effective psychotherapeutic intervention would be a tremendous improvement of mental health care.

Several attempts have been undertaken to predict sad mood in winter based on psychological examinations or biomarkers measured in summer. The best indicator for a likely occurrence of depression in winter is the individual's report on prior experience of seasonal symptoms, e.g., according to the seasonal pattern assessment questionnaire (SPAQ, 14). The SPAQ is still the most used instrument for estimating subjective experience of seasonal occurrence of depression symptoms. Furthermore, psychological research has considered cognitive vulnerabilities. In patients with SAD, there is a bias toward remembering words of negative valence more likely in the winter than in the summer (15). In addition to remembering negative words more likely, patients with depression also create more false memories than healthy controls and perceive even positive items with a less positive, i.e., more negative valence (16). Individuals with SAD estimate future negative events as more likely to happen (17), and demonstrate a high level of automatic thoughts and dysfunctional assumptions (18) as well as negative attributions (19). Such psychological features, i.e., cognitive-behavioral factors such as increased rumination, automatic thoughts and dysfunctional attitudes were shown to be not only indicative (20) but even predictive for SAD (21, 22). A ruminative response style as measured in fall predicts symptom severity in winter (21, 22) which indicates a predisposition for ruminative processes being mediators for SAD symptomatology. When examining rumination, it is crucial to distinguish between trait and state rumination (23), as induced state rumination predicts negative affect, independent of the extent of trait rumination (24). Recent theoretical accounts (25) and empirical evidence also suggest that increases in state negative affect may subsequently set of increases in state ruminative thinking in an automatic and habit-like way, and are associated with symptoms of depression and depression status (26, 27). Emotional responses were also combined with attention demands in an emotional Stroop task to predict subsequent levels of symptomatology with tests in winter and follow-up in summer (28). Performance in the Stroop task relies on cognitive flexibility, and cognitive flexibility is impaired in depression (29, 30).

Biomarkers have mainly been derived from major biological hypotheses regarding circadian rhythms, neurotransmitters, and molecular genetics (31). Circadian rhythms were suggested to play a role in SAD, where according to the phase-delay hypothesis the patient's circadian rhythm is delayed relative to the daily routine of sleeping/resting and waking/activity (4). Well in line with this hypothesis, SAD is especially common in younger subsamples who are often evening chronotypes, and in general in people with evening chronotype (32). Moreover, in patients with SAD, depressive symptoms are typically worse in the morning (33).

Another approach to identify vulnerability to SAD and, thus, find predictive biomarkers is based on neuroimaging. Since the brainstem is affected by photoperiodic changes, a large study used magnetic resonance imaging to determine a relation between brainstem volume and low mood (34). In this study, a relationship between photoperiod, volume of whole brainstem, pons and medulla, and low mood and anhedonia was found only in women, but not in men. Women with the short 5-HTTLPR genotype who suffered from SAD showed higher 5-HTT levels compared to those who did not suffer from SAD in the ventral striatum, right orbitofrontal cortex, middle frontal gyrus, left supramarginal gyrus, left precentral gyrus, and left postcentral gyrus and this difference was most pronounced during winter (35).

However, as neuroimaging and genetic testing is not widely available, the most convenient approach to predict SAD is a psychological examination. In order to boost accuracy of prediction a physiological marker could be added that is easily obtained at low cost. The electroencephalogram (EEG) is a method that is traditionally used in clinical and research settings, but commercial products for brain computer interfacing, e.g., in the gaming industry raise the hope that soon there will be easy-to-use systems available that can combine the lightweight design of devices used in non-professional settings with the accuracy needed for clinical and research questions. Indeed, it was demonstrated by a limited number of EEG studies in northern countries that EEG-biomarkers correlate with the absence of daylight and with midnight sun, and factors such as responsiveness of the brain to lighting conditions but also sleep were discussed to be the source of this variance (36, 37). The EEG is also indicative for depression and variants of it (38, 39). The earlier mentioned valence effects of memorized visual stimuli are detectable in the EEG (40), further suggesting top-down attentional modulation of emotional memory bias. Broadband lower absolute EEG-power was found in patients with major depression disorder (41), but especially in the theta (42, 43) and alpha frequency band and especially in the frontal cortex (41, 44–47). The prefrontal cortex is also involved in rumination (48–50). Interestingly, beta and alpha power varies with seasons (51, 52), and so does frontal alpha asymmetry (53). Abnormalities in beta and alpha power as well as frontal alpha asymmetry are also specific for SAD (54–58).

In this study, we aim to identify biomarkers in the EEG which, when measured in summer, allow prediction of increased depressive symptoms in winter. We aim to combine these biomarkers with personal characteristics, depressive symptoms, emotional reactivity (mood induction in an experimental task), and cognitive vulnerabilities such as rumination tendencies, thoughts and beliefs, and habitual characteristics of negative thinking to answer the question whether it is beneficial to add EEG biomarkers to prediction models for depressive mood in winter.

## 2. Methods

### 2.1. Ethics

We obtained prior approval from the Icelandic National Bioethics Committee on May 28th 2019 (study number 19-090-V1). All investigators signed a non-disclosure contract and written informed consent was obtained prior to inclusion from all participants.

### 2.2. Research Setting

The study was carried out as a collaboration between the University of Akureyri and the University of Iceland. The baseline assessment was performed between July and September 2019 in the EEG laboratory of the Faculty of Psychology at the University of Akureyri. Follow-up assessments were conducted in October, January, and April 2020 *via* online questionnaires and telephonic reminders conducted by the team at the Faculty of Psychology, University of Iceland. For the purpose of the present manuscript, only data from the follow-up in January was analyzed.

### 2.3. Recruitment

Participants were recruited *via* email to students at the University of Akureyri, as well as *via* advertisement in social media, directing interested individuals to a webform. Inclusion criteria were the minimum age of 18 years, proficiency in Icelandic, and the ability to give informed consent for participation. For completion of all follow-ups participants were remunerated with a voucher of 4,000 ISK for a local shop.

### 2.4. Procedure

Baseline assessment took about 120 min. After participants completed informed consent, a digital questionnaire, consisting of 72 custom made questions and the questionnaires as listed in Section 2.5 were answered by the participants. While participants answered the questions, the EEG was mounted with electrolyte containing a mild abrasive in order to achieve impedances below 10 kΩ. Before recordings began participants were shown the effect of muscle movement on the EEG and consequently instructed to keep movements to a minimum and asked to refrain from talking during the recordings.

The first two conditions were resting state measurements which lasted for 3 min, with eyes open and eyes closed respectively, and with the computer screen turned off. The other tasks were presented on a screen based on the Psychtoolbox in Matlab. First, in the emotional picture learning task participants were shown 60 pictures from the OASIS database (59), balanced for negative, neutral, and positive valence and low, medium, and high arousal. Participants were informed that in the subsequent task they would be asked to recall the pictures shown. The task required to indicate whether each picture represented spring, summer, fall, or winter by pressing a corresponding key on the keyboard with the right hand to ensure attention and to prime seasonal concepts. Pictures were shown with an inter-trial interval of 1 s and a variance of 0–10 screen flip intervals during which a fixation cross was presented. All pictures were shown for at least 2 s and otherwise until participants responded *via* key press. In the following the picture recall task participants were asked to freely recall and name pictures seen in the previous task. Their answers were noted by the experimenter. Subsequently, a recognition condition involved presentation of the pictures from the picture learning task but randomly intermixed with 60 new images, again balanced for valence and arousal. Participants were asked to indicate *via* keys on the keyboard whether each picture was new or previously seen. Timing of the presentation was the same as in the learning condition. The next condition was a Stroop task where participants were asked to indicate the font color of words displayed on the stimulus computer by pressing a correspondingly colored key on a keyboard. There were 105 congruent trials and 210 incongruent trials, presented in a randomized order, with an inter-trial interval of 1 s and a variance of 0–10 screen flip intervals during which a central fixation cross was presented.

In the final condition, the rumination task, participants received a printed three part form containing questions about their current emotional state and the brief state of rumination inventory (BSRI, 23). All instructions were given verbally through headphones and partly additionally on a screen. Firstly, participants completed part A on the form containing one question on their current emotional state and the 8-item BSRI. Next, an 8 min musical piece was played in order to evoke temporary sadness or dysphoria, and participants asked to freely experience any emotions they might feel. We used a musical excerpt from Prokofiev's “Russia Under the Mongolian Yoke,” remastered at half speed. Prior research has shown that this approach can effectively cause a transient dysphoric mood (60–62).

Immediately after the song had finished, participants answered the forms' part B containing one question on their current emotional state. They were then instructed to wait in silence for 5 min for a challenging cognitive task. However, no cognitive task followed but the waiting period served as a free contemplation time in anticipation of a task. In the third and final part of the rumination task participants answered an 8-item BRSI and one question on their current emotional state.

On the day following the EEG recording, participants began the baseline measurement of the studies follow-up phase which consisted of a 4 day long measurement period using the mobile application ExperienceSampler (63) with which mood fluctuations over the course of a day along with activity level, fatigue, and rumination was assessed in questionnaire form. Five measurements were taken at random times each day between 9:00 a.m. and 21:00 p.m.

The three subsequent follow-up intervals, conducted in October, January, and April, had the same form as the baseline measurement with the addition of a 48 question internet survey. The internet survey consisted of the following questionnaires: Patients Health Questionnaire, Rumination Responses Scale-short form, Perceived Stress Scale, and Depression Anxiety Stress Scales (see Section 2.5 for more details). In addition, the survey included questions on recent traveling and use of any depression treatments.

### 2.5. Questionnaires

We assessed subjective perception of mood and behavioral change with seasons with the seasonal pattern assessment questionnaire (SPAQ, 1). It includes 8-items regarding seasonal change in mood and behavior, pattern of seasonal change, reactivity to different climatic and atmospheric conditions and whether and to what extent those changes affect the individual (64). We used the Icelandic version, which performed compared to a diagnostic clinical interview with a sensitivity of 94%, a specificity of 73%, and a combined positive predictive value of 45% for SAD and subsyndromal SAD (65). The questionnaire is acknowledged as an effective screening tool for SAD, with an internal consistency of α=0.74–0.81 and a test-retest reliability of 0.76 at an interval of 2 months.

As mentioned in Section 2.4, we examined state rumination before and after mood induction with the 8-item BSRI (23). The Ruminative Responses Scale-short form (RRS, 66) was used to measure the degree of trait rumination.

The habit index of negative thinking (HINT, 67) measures in 12 items habitual characteristics of negative thoughts (i.e., automaticity, lack of intent and awareness, difficult to control). In addition, we measured mood with the Patient Health Questionnaire (PHQ, 68), sleep problems with the Bergen Insomnia Scale (BIS, 69), depression, anxiety, and stress with the Depression Anxiety Stress Scale (DASS, 70), positive attitudes toward ruminative thinking with the Positive Beliefs in Rumination Scale (PBRS, 71), chronotype by using the Morningness Eveningness Questionnaire—Revised (MEQ-R, 72), and to what extent people were following habits with the Creature of Habit Scale (COHS, 73). Participants were also asked about their age, gender, handedness, first language, body weight, and height from which we calculated the body mass index (BMI).

Furthermore, we asked about optimism, nutrition, mental or neurological diseases, regularly taken medication, current tiredness, bed- and waketime the night before the experiment, exercise, phase of menstrual cycle in women, and weather, but we did not include the respective data into the present manuscript.

All questionnaires and written materials used in this study were in Icelandic, therefore, only participants who were fluent in Icelandic were recruited for the study. This was ensured by having all recruitment material in Icelandic. In Iceland, 96% of the population speaks Icelandic, with 12.4% of the population being foreign citizens in 2019 according to Statistics Iceland (www.statice.is).

### 2.6. EEG Recording and Preprocessing

EEG data was recorded with software and hardware from Brain Products GmbH (Gilching, Germany) at a sampling rate of 1,000 Hz with an EasyCap in an extended 10-20 system, including 32 electrodes (Fp1, Fp2, F3, F4, C3, C4, P3, P4, O1, O2, F7, F8, T7, T8, P7, P8, Fz, Cz, Pz, FC1, FC2, CP1, CP2, FC5, FC6, CP5, CP6, FT9, FT10, TP9, TP10) referenced to FCz and grounded at AFz. In addition, lower vertical electrooculogram was recorded.

EEG-data was pre-processed with BrainVision Analyzer (Brain Products GmbH, Gilching, Germany). First, band-pass filters from 0.5 to 48 Hz with zero-phase shift Butterworth filters were applied. Then, data was re-referenced to common average. Next, an independent component analysis (ICA) was used such that in the backtransform the signals that include eye-blink artifacts would be removed (infomax restricted algorithm). Finally, remaining artifacts were identified and excluded automatically by the following standard thresholds: check gradient (maximal allowed voltage step: 50 microvolt/ms), check difference (maximal allowed difference of values in intervals of 200 ms: 200 microvolt), lowest activity allowed in 100 ms intervals: 0.5 microvolts. The artifacts that were identified in this way were excluded with a time-range of ±200 ms.

The EEG recorded during 3 min of rest with eyes open, 3 min rest with eyes closed, the whole recall session, and the last 3 min out of 5 min of sad mood induction was segmented into equal-sized epochs of 2 s. From the learning and recall conditions 1 s starting at stimulus presentation were extracted, and they were processed separately for negative, neutral, and positive pictures for learning, and in addition for old and new pictures for recognition. From the Stroop task 500 ms from stimulus onset, and congruent and incongruent conditions were processed separately. Thus, in total, there were 15 conditions extracted from the EEG experiment that were submitted to feature extraction.

### 2.7. Feature Extraction

For all of these conditions and each segment we extracted features based on the multivariate autoregressive model with the functions mvfreqz.m and mvar.m from the BioSig toolbox (74) with model order 10, and partial correlation estimation with unbiased covariance estimates (75), which is an accurate estimation method (76). The multivariate parameters in the frequency domain that can be derived from these transfer functions were computed for 1 Hz frequency steps between 1 and 48 Hz. Only data from electrodes F3, F4, C3, C4, P3, P4, O1, O2, F7, F8, T7, T8, P7, P8, Fz, Cz, Pz was used. The measures that were extracted were the following:

- **Spectrum:** The auto- and the cross-spectrum, which is the Fourier transform of the cross-covariance function (77).
- **Direct causality:** Direct causality as developed by (78); this measure is not computed for each frequency.
- **Transfer function:** Related to the non-normalized directed transfer function (79).
- **Transfer function polynomial:** Frequency transform of a polynomial describing the transfer function. It is related to coherence as the absolute of the squared transfer function polynomial represents the non-normalized partial directed coherence (79).
- **Real valued coherence:** The real part of the complex-valued coherence (80) is an ordinary coherence (74).
- **Complex coherence:** The imaginary part of the complex-valued coherence (80).
- **Partial coherence:** Designed by (81) it's concept is that one channel drives the other channels if the first channel explains or accounts for the linear relation between the other two.
- **Partial directed coherence:** An extended concept of partialized coherence, measuring the relative strength of the direct interaction between pairs of signals (82).
- **Partial directed coherence factor:** An intermediate step between partial coherence and partial directed coherence by adding directionality to partial coherence and including instantaneous causality (82).
- **Generalized partial directed coherence:** In contrast to partial directed coherence, generalized partial directed coherence is invariant against scaling differences between signals (83, 84).
- **Directed transfer function:** Represents information that flows from one region to another over many possible alternative pathways (85).
- **Direct directed transfer function:** Extends directed transfer function by separating direct from indirect causal relations of signals (86).
- **Full frequency directed transfer function:** In contrast to directed transfer function, the full frequency directed transfer function is normalized with respect to all the frequencies in the predefined frequency interval (86).
- **Geweke's Granger Causality:** A bivariate version (87) of Geweke's Granger Causality (88).

Finally, we also included the power-spectral density as a feature, representing band-pass power in 1 Hz frequency steps from 1 to 48 Hz.

### 2.8. Features and Feature Combinations for Machine Learning

For classification, we considered three situations:

- EEG features only; each EEG feature was used individually, i.e., we conducted for each of the 15 conditions classification with each of the 16 feature vectors as described in Section 2.7.
- Questionnaire data only; We classified participants by a feature vector including their total scores in PBRS, the three mood measurements in the mood induction task individually and also the difference between the first two and the latter two, and the two rumination measurements with the BSRS in the mood induction task, SPAQ global seasonality score, HINT, DASS stress and anxiety, RSS brooding and reflection, sex, age, education, body mass index (BMI) calculated by the participants indication of height and weight, MEQ, BIS, and COHS.
- A combination of each of the EEG features and conditions with the questionnaire feature vector.

### 2.9. Grouping for Prediction Modeling

For machine learning we divided the sample into the group experiencing depressive symptoms in winter and a control group. For defining the borders between these groups we used the diagnostic criteria according to the DASS-21 subscale for no depression, mild depression, and moderate depression (70). The control group included participants which were not depressed at baseline in summer as well as at winter follow up in January, i.e., showing <10 points on the DASS-21 depression scale at both timepoints. For prediction we did two analyses, for mild and moderate decline of mood, i.e., increase of depressive symptoms. Both groups showed no depression at baseline (DASS-21 depression scale <10). The group with at least mild increase of depressive symptoms comprised those participants who would show a depression score on the DASS-21 of at least 10 points at follow up in January. The group with moderate increase of depressive symptoms comprised those participants who would show a depression score on the DASS-21 of at least 14 points at follow up. Thus, the group with at least mild increase of depressive symptoms overlapped with the group of at least moderate increase of depressive symptoms.

### 2.10. Machine Learning and Statistical Analysis

We used leave-one-out cross-validation, thus, a model was fitted for each participant to all participants but the left-out participant using the Matlab function *fitclinear* using a logistic regression as learner. The fitting procedure was performed with a regularization term strength λ of 10^−11^.We used lasso (L1) penalty for the composition of the objective function for minimization from the sum of the average loss function, with sparse reconstruction by Separable Approximation (SpaRSA) as objective function minimization technique and 10^−8^ as gradient tolerance. The initial linear coefficient estimates were set to zeros as initial values and the learning rate was constant.

Lasso regularization reduces the number of predictors, identifies important predictors and selects among redundant predictors, which is important in the high-dimensional feature space of EEG biomarkers extracted with the multivariate autoregressive model. As λ increases, the number of nonzero components of β increases. Intuitively, the predictor coefficients β are therefore indicative for each feature's importance to the model and were therefore reported graphically with the results to demonstrate which brain regions/frequency range contributed most to the prediction of worsening of depressive symptoms in winter.

Machine learning results were gathered overall as accuracy (% of correctly classified individuals overall), specificity (% of correctly classified individuals who did not show depressive symptoms in winter, i.e., correctly classified controls), sensitivity (% of correctly classified individuals with depressive symptoms in winter), positive predictive value (PPV; % of predicted cases actually developing depressive symptoms in winter), and negative predictive value (NPV, % of not predicted actually not developing depressive symptoms in winter).

For psychological self-report questionnaires we calculated means and standard deviations separately for controls and the overall group of people experiencing mild or moderate decline of mood in winter, as well as Mann–Whitney *U*-tests comparing these two groups at baseline. A non-parametric test was chosen because the questionnaire data is ordinal, so parametric tests should not be used.

## 3. Results

### 3.1. Sample

A total of 119 participants were recruited for this study and participated in the baseline assessment in summer 2019. Among them, 89 participated in the second follow-up in winter (January 2020). After exclusion of missing data in four participants, 18 participants showed no depression at baseline and mild worsening from baseline to follow up, and 11 among those showed moderate worsening of depression from baseline to follow-up. The control group of 46 participants was free of depression at baseline as well as at follow up.

The control group sample consisted of 40 women and 6 men, while the group of participants with at least mild increase of depressive symptoms consisted of 16 women and 2 men. The odds ratio for gender to suffer from SAD is 1.8 according to (89) justifying an overall overrepresentation of female participants in our sample.

In the sample of controls/participants with at least mild worsening of depressive symptoms in winter, 8.70/0% had completed primary education, only 47.83/50% had higher education entrance qualification, 2.17/11.11% had learned a trade, 28.26/22.22% had completed undergraduate education at a university, and 10.87/11.11% had completed master or doctoral level education at a university. The native language was Icelandic in 95% of the sample, however, all participants were fluent in Icelandic.

Descriptive statistics for the self-report questionnaires, separately for the two groups as well as results from Mann-Whitney U-tests comparing the two samples are shown in Table 1. For some measures there is evidence that the groups are very inhomogenous. For chronotype, as measured by the MEQ, in the group experiencing at least mild depressive symptoms, the standard deviation is twice the mean, whereas for symptoms of insomnia, as measured by the BIS, the standard deviation is even three times as large as the mean.

**Table 1:** Self-reported characteristics of the control group and group with worsening of depressive symptoms in winter at baseline.

	**Controls**		**Depressive symptoms**		***U*** **-test**	
**Scale**	**Mean**	**SD**	**Mean**	**SD**	**z**	***p***
Age	33.61	18.21	31.61	16.92	0.11	0.91
BIS	−20.13	25.52	−10.03	34.37	−1.43	0.15
BMI	26.06	6.79	24.82	11.37	−0.01	0.99
BSRI t1	219.52	154.05	287.25	182.97	−1.32	0.19
BSRI t3	228.56	191.93	285.8	221.62	−0.72	0.47
COHS	78.46	21.26	75.06	42.92	−1.33	0.18
DASS anxiety	2.87	4.23	8.56	6.78	−3.59	<0.001
DASS depression	2.87	2.72	3.67	3.01	−0.93	0.35
DASS stress	8.3	6.42	15	8.35	−2.88	<0.001
Education	2.8	1.36	2.78	1.31	−0.14	0.89
GSS	5.57	4.75	9.94	4.45	−3.26	<0.001
HINT	29.67	17.22	54.5	17.02	−4.22	<0.001
MEQ	35.24	36.08	21.04	41.98	1.16	0.25
Mood t1	109.95	34.35	103.98	23.21	1.46	0.14
Mood t2	77.24	38.6	82.94	41.38	−0.49	0.63
Mood t3	95.08	35.22	92.97	27.78	0.43	0.67
PBRS	23.11	6.27	24.33	3.94	−0.84	0.4
PHQ	4.11	3.09	7.83	3.55	-3.63	<0.001
RRS brooding	8.04	2.62	9.78	3.84	−2.08	0.04
RRS reflection	8.2	3.11	9.89	2.93	−2.01	0.04

*BIS, Bergen insomnia scale; BMI, body mass index; BSRI, brief state rumination inventory; t1, before mood induction; t3, after mood induction; COHS, creature of habit scale; DASS, depression, anxiety, stress scales; GSS, global seasonality score; HINT, habit index for negative thinking; MEQ, morningness-eveningness questionnaire; mood, emotional state; t2, after sad music; PBRS, positive believes in rumination scale; PHQ, patient health questionnaire; RRS, ruminative response scale; SYM, group with at least mild depressive symptoms in winter*.

### 3.2. Classification Results

**Figure 1:**
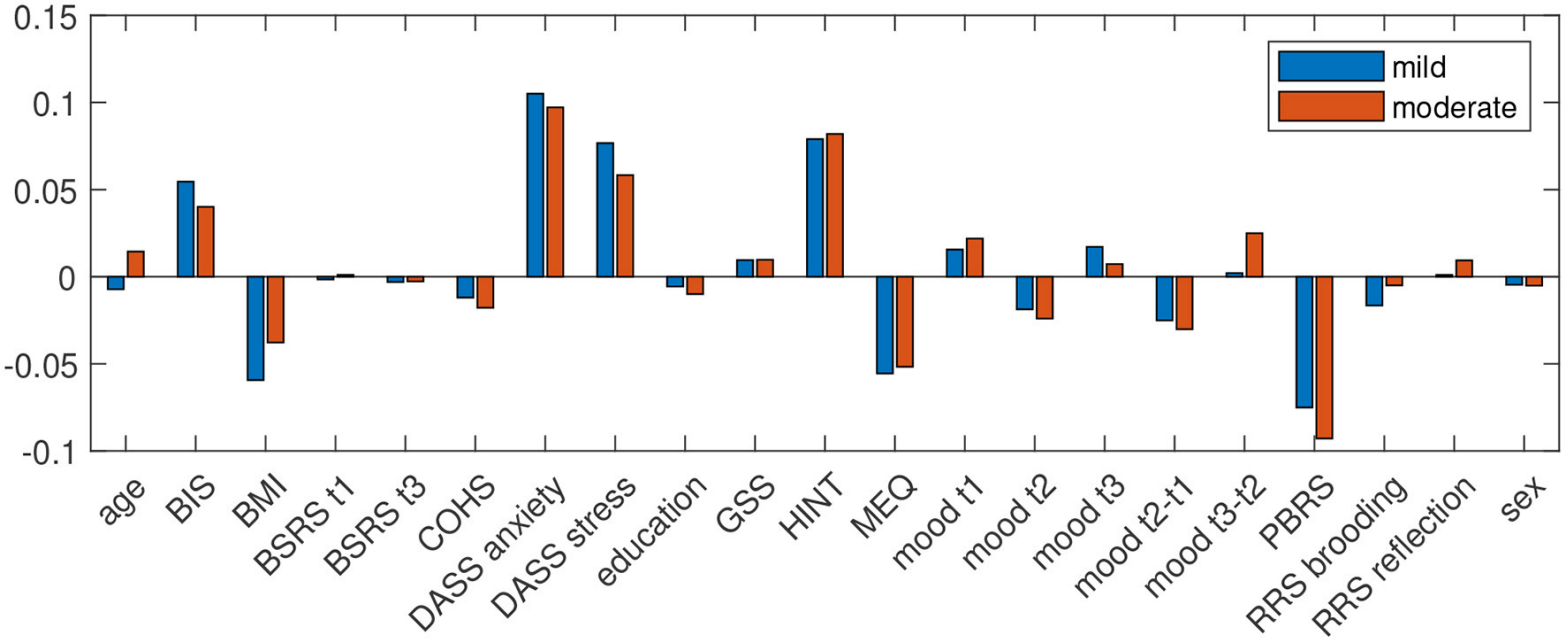
Predictor coefficients β for each questionnaires' importance to the model for the prediction of worsening of depressive symptoms in winter to at least mild (DASS depression score ≥10; blue) and moderate (DASS depression score ≥14; red) extent.

For questionnaire data, only, accuracy was 76.56 for prediction of mild and 82.46 for moderate depressive symptoms in winter, with a specificity (accuracy to classify control group participants correctly) of 78.26 and 84.78 and sensitivity (accuracy to identify participants who experience depressive symptoms in winter) of 72.22 and 72.73. Positive predictive value (PPV) for at least mild depressive symptoms in winter was 56.52 and for moderate depressive symptoms it was 53.33. Negative predictive value (NPV) for at least mild depressive symptoms in winter was 87.80 and 92.86 for moderate depressive symptoms in winter. Figure 1 shows the predictor coefficients β for each feature's importance to the model for the prediction of worsening of depressive symptoms in winter for the two classifications. Relative importance of features were highly consistent for the two prediction models. Predictive for worsening of depressive symptoms in winter were low quality of sleep, low BMI, anxiety, stress, habits of negative thinking, eveningness, and a low degree of positive believes in rumination, measured in summer.

When using EEG data, this resulted in 15 times 16 classifications for each condition and each feature used. EEG alone for prediction of mild depressive symptoms in winter yielded best classification accuracy for partial directed coherence factor extracted during the Stroop task's matching condition (accuracy: 71.88, specificity: 80.43, sensitivity: 50). Because sensitivity was at guessing level it is safe to not interpret these results any further. PPV for at least mild depressive symptoms in winter was 50, NPV was 80.43.

**Figure 2:**
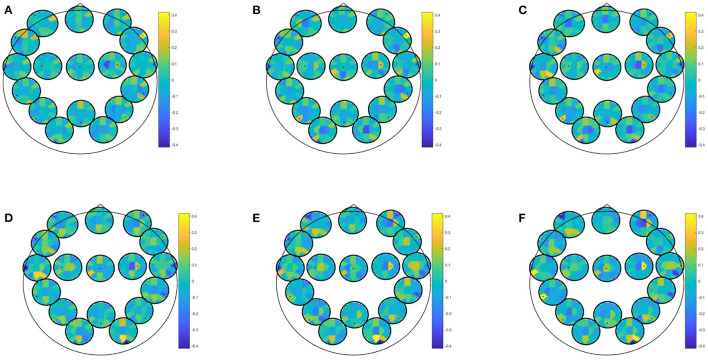
Predictor coefficients β for directed transfer function (DTF) extracted during recognition of positive pictures. Coefficients indicate importance of the feature to the model for prediction of at least moderate depressive symptoms in winter. **(A)** Delta 1–4 Hz, **(B)** Theta 5–7 Hz, **(C)** Alpha 8–12 Hz, **(D)** Beta 13–20 Hz, **(E)** Beta 2 21–30 Hz, **(F)** Gamma 31–48 Hz.

EEG alone for prediction of moderate depressive symptoms in winter yielded best classification accuracy for directed transfer function during recognition of previously seen positive pictures (accuracy: 82.46, specificity: 97.83, sensitivity: 73.33%), PPV for at least moderate depressive symptoms in winter was 88.99, NPV was 93.88. Predictor coefficients β shown in Figure 2, showing which frequencies and brain connections were most predictive. The directed transfer function showed a broader network with interhemispheric frontal connections in the delta to theta range, frontocentral connections in the alpha-gamma range, and temporo- and fronto-occipital connections in the alpha-gamma range. Autocorrelations were also identified to be predictive right frontal, central, bilateral temporal, and right occipital in the alpha-gamma range.

**Figure 3:**
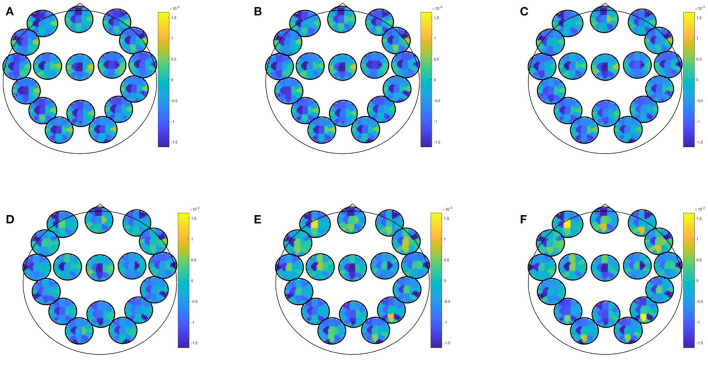
Predictor coefficients β for questionnaire data combined with directed transfer function (DTF) extracted during rest with eyes open. Coefficients indicate importance of the feature to the model for prediction of at least mild depressive symptoms in winter. **(A)** Delta 1–4 Hz, **(B)** Theta 5–7 Hz, **(C)** Alpha 8–12 Hz, **(D)** Beta 13–20 Hz, **(E)** Beta 2 21–30 Hz, **(F)** Gamma 31–48 Hz.

EEG combined with questionnaire data yielded best classification accuracy for prediction of at least mild depressive symptoms in winter with an accuracy of 81.25 (specificity: 82.61; sensitivity: 77.78; PPV: 63.64; NPV: 90.48). This result was obtained by most biomarkers (i.e., partial directed coherence, coherence, directed transfer function, direct directed transfer function, full frequency directed transfer function, partial coherence, partial directed coherence factor, generalized partial directed coherence, and power spectral density) and most conditions (i.e., EEG data recorded during eyes closed or open, learning of neutral or positive images, recognition of recognition of new positive pictures, recognition of previously seen neutral or positive pictures, rumination, Stroop match and non-match condition). Predictor coefficients β for directed transfer function are given in Figure 3 extracted during rest with eyes open. Again, frontal and central regions were strongly involved, across all frequency ranges. Most of the frontal involvement was autocorrelative, except for a frontocentral correlation in the higher beta and gamma range. Central correlation was highly pronounced for central-left area to all other areas in all frequency bands.

**Figure 4:**
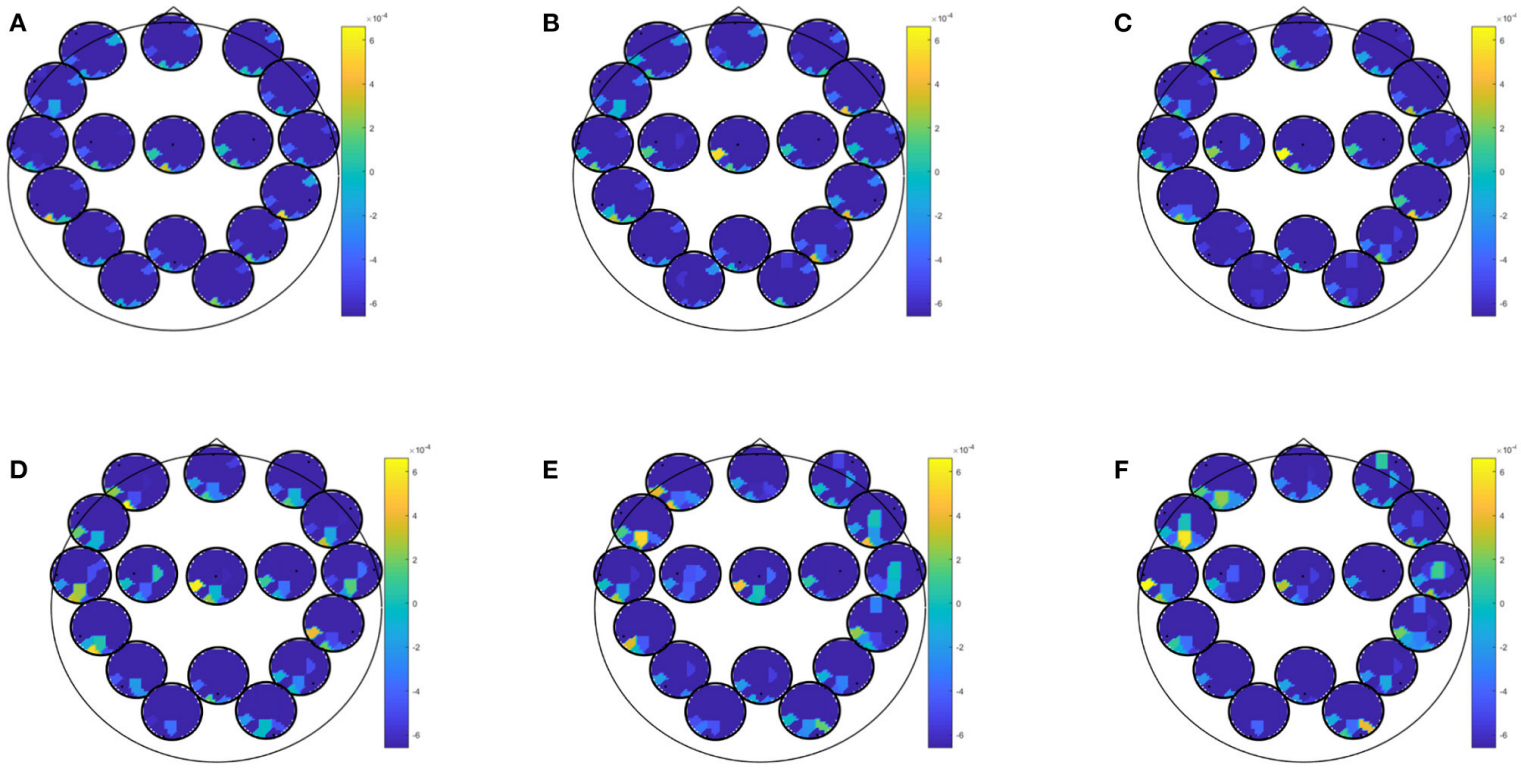
Predictor coefficients β for questionnaire data combined with directed transfer function (DTF) extracted during rumination. Coefficients indicate importance of the feature to the model for the prediction of at least moderate worsening of depressive symptoms in winter. **(A)** Delta 1–4 Hz, **(B)** Theta 5–7 Hz, **(C)** Alpha 8–12 Hz, **(D)** Beta 13–20 Hz, **(E)** Beta 2 21–30 Hz, **(F)** Gamma 31–48 Hz.

EEG combined with questionnaire data yielded best classification accuracy for prediction of at least moderate depressive symptoms in winter with an accuracy of 85.96 (specificity: 86.96; sensitivity: 81.82; PPV: 60.01; NPV: 95.24). This result was obtained by several biomarkers (i.e., spectrum, partial directed coherence, directed transfer function, partial coherence, generalized partial directed coherence) and most conditions (i.e., EEG data recorded during eyes open, learning of negative, neutral or positive images, recognition of new positive pictures, rumination, Stroop match, and non-match condition). Predictor coefficients β for directed transfer function are given in Figure 4, showing where in the brain oscillatory activity in a certain frequency band was most predictive for directed transfer function during rumination. Most areas were highly involved in this prediction, but fronto-occipital and temporo occipital connections on the left hemisphere dominated the higher beta-gamma range.

## 4. Discussion

In this study we aimed to identify biomarkers in the EEG and self-reported characteristics which, when measured in summer, can be used for accurate prediction of whether an individual will suffer from worsening of depressive symptoms in winter. We found that indeed, a combination of cognitive aspects and EEG biomarkers allows for a better prediction than the cognitive aspects or EEG biomarkers alone. Prediction accuracy was better for prediction of at least moderate depressive symptoms as compared to mild depressive symptoms, which could be due to the clearer distinction of the group with at least moderate depressive symptoms from the control group. However, since these two groups overlap, and since the statistical power is limited for the group with at least moderate depressive symptoms we limit the discussion to the sample of at least mild depressive symptoms in winter.

### 4.1. Self-Reported Characteristics and Cognitive Vulnerabilities

We found that low quality of sleep, low BMI, anxiety, stress, habits of negative thinking, eveningness, and a low degree of positive believes in rumination, measured in summer predicted worsening of depressive symptoms in winter.

Insomnia or, more generally, sleep problems were related to SAD in earlier studies (5, 32, 90–96). In our sample, the variance of symptoms of insomnia was very high in the group who would develop at least mild depressive symptoms in winter, suggesting that there might be different subgroups, reflecting different paths of vulnerability that is based on the different factors causing insomnia, being either physiological or cognitive-behavioral in nature. On the one hand, increased rumination is linked to insomnia (97), but on the other hand, insomnia might be the symptom of a physiological vulnerability. It has been suggested that in some cases, insomnia could be treated by administration of melatonin (98), and there have also been attempts to treat SAD by melatonin (99, 100). Further evidence links melatonin to the serotonergic system, which in turn, is linked to depression, anxiety, and stress (101). Melatonin treatment alters the expression of genes of serotonergic neurotransmission in a mouse model of SAD (102). More evidence points to involvement of major monoamine neurotransmitters serotonin, norepinephrine, and dopamine in SAD (103). Therefore, biochemical markers such as cortisol awakening response as a marker for hypothalamic-pituitary-adrenal axis function (104) and serotonin-transporter binding (105) have been suggested. In line with the daily rhythm of cortisol, eveningness was found previously to be strongly related to seasonality and SAD (32, 95, 96). Being a serotonin-transporter-linked polymorphic region (5-HTTLPR) short allele carrier was found to be a risk factor for developing SAD (1). A genotype-dependent increase in winter of serotonin transporter binding was found to be specific for patients with winter depression (105). However, although the level of serotonin transporter binding is comparable between healthy controls and patients with winter depression during summer, the patient group shows a lower increase from summer to winter as compared to controls (105). The link to the serotonergic system is supported by predictability of relapse during winter based on depressive symptoms during tryptophan depletion in summer (106). Possibly related to neurotransmitter systems, low vitamin D3 levels where also suggested to predict depressive symptoms increase from fall to winter (107).

The fact that melatonin was suggested as a treatment for SAD (99, 100) is closely linked to the finding that melatonin levels underly a strict circadian rhythm. Administrating melatonin at a specific time of the day triggers a change in sleeping time, which can help to ameliorate the so-called social jetlag of the evening chronotype (108). As chronotype changes with age toward more morningness, the vulnerability for anxiety and depression also decreases (109). Also in our sample, the late chronotype was predictive for depressive symptoms in winter, although the group showed a large variance in the respective score. This detail could point toward considerable inhomogeneity of the group in terms of their chronotype. Although being an evening chronotype is a risk factor for developing depressive symptoms in winter, also individuals reporting to be morning or neutral chronotypes in summer might suffer from mood decline during the dark season. Conclusions to be drawn on this end are limited, but it was also reported that chronotype self-reports do vary with the season especially in individuals with winter depression more morningness was measured in the summer than in the winter (110).

Although controls and individuals who would develop at least mild depressive symptoms in winter did not differ significantly by BMI in summer, the highly negative beta values as indicated in Figure 1 suggest that a low BMI in summer can serve as a predictor for worsening of depressive symptoms in winter. The similarity in BMI between the two groups in summer indicates that the relationship between BMI and vulnerability to seasonal mood fluctuations is not too large. The relation between body weight and SAD has been investigated previously. A higher BMI at baseline was found to predict treatment outcome of 6 weeks light treatment (111). As emotional eating and weight gain are associated with SAD (112), our finding seems to be unexpected. However, it was shown recently that the self reported seasonal changes in weight is related to lower plasma adiponectin levels, an indicator for metabolic dysregulation (113). Therefore, it might be that the difference in weight between summer and winter is more relevant, and a lower BMI in summer might be indicative for a larger weight gain. However, this is pure speculation and needs to be addressed in future studies.

Habits of negative thinking were assessed with the HINT, where negative thoughts are characterized by automaticity, lack of intent and awareness, and difficulty to control them (67). HINT scores predicted mood worsening in winter, whereas trait ruminative brooding and reflective pondering did not. This may suggest that people experiencing their self-focused negative thoughts as being triggered more automatically and without intent, awareness, or control, are specifically vulnerable to experience upward shifts in symptoms of depression during winter.

Individuals with SAD also seem to estimate future negative events as more likely to happen (17), which is a concept closely related to the habit of negative thinking as well as the optimism-pessimism as assessed in our study. Endorsement of emotional adjectives and a negative attributional style are elevated in patients with SAD (114). However, in contrast to our data it was previously reported that these cognitive aspects could not be used to predict later symptom levels (114). Also, countering our expectations, a lower score in summer on the PBRS indicated a higher risk for worsening of depressive symptoms in winter. Prior research showed that rumination is linked to depression (115) and that positive beliefs about rumination are associated with ruminative thinking, mediating further a negative association with positive affect (116). While prior research suggests that increased rumination are predictive for SAD (21), we could not confirm this relationship. In our data, a negative predictor coefficient for the PBRS suggested that positive beliefs in rumination would rather protect from worsening of depressive symptoms in winter. This finding is difficult to explain but warrants further investigation. The first four items of the Icelandic translation (e.g., “I need to consider things to realize how I feel.”) of the scale could have been rather interpreted as being indicative for a positive attitude toward being considerate, which might indeed be a protective factor instead of a risk factor.

Furthermore, patients with prior experience of SAD show depressive affect in response to low light intensity stimuli (21), another indicator for emotional responses to darkness. However, as this result is based on prior experience of SAD, it might rather be due to reactivated memories of sad mood during the dark period rather than an indicator for emotional response style. In line with the potential role of memory mechanisms, autobiographical memory style was examined in winter in individuals with SAD (117). It was found that the number of overly general memories that were generated in response to positive cues was related to symptom levels measured during remission in summer (117).

### 4.2. EEG Biomarkers

Best results for EEG alone were of guessing level for sensitivity for the prediction of mild depressive symptoms in winter, which might indicate that a mild increase of depressive symptoms can not be predicted by EEG features.

EEG alone yielded sensitivity at guessing level for prediction of mild depressive symptoms in winter, which should, therefore, not be interpreted any further.

The frontal involvement and EEG-results in general were more reliable when EEG was combined with self-report questionnaires, emphasizing further the superiority of self-report data over EEG biomarkers. Specifically, the high stability across biomarkers and conditions indicates that EEG combined with self-report data can contribute reliable additional information, but the consideration of the self-report data is crucial.

EEG studies have identified some likely structural and activational irregularities being candidates for the neurological mechanisms involved in depressive tendencies and depressive mechanisms such as rumination. For example, lowered alpha activity in the prefrontal cortex is thought to predict higher tendency to ruminate (50). Inefficient information transfer from the left dorsolateral prefrontal cortex to the temporal lobe structures might be critical for trait rumination (48). The involvement of the frontal cortex as well as the alpha frequency range points to the role of cognitive control over negative thoughts. High alpha power is acknowledged to reflect active inhibition (118). Therefore, the involvement of alpha activity in the left hemisphere can be interpreted as reduced cortical activity. It was theorized that hypoactivation of the left frontal area leads to ruminative tendencies and consequently to negative emotional interpretation (119). The frontal cortex is also involved in cognitive flexibility (120), which has been reported to be impaired in individuals with depression (29). Specifically, in negative emotional contexts individuals with major depressive disorders were suggested to exhibit ruminative and negative automatic thoughts because they lack cognitive flexibility (30).

An abnormal activation in the lower left frontal cortex has been found to be critical regarding depressed individuals' tendency to pay greater attention to adverse stimuli (41, 47). Abnormalities in the activation or structure of the circuitry of emotion, which includes the prefrontal cortex, anterior cingulate cortex, hippocampus and amygdala have been suggested to underlie depressive disorders (121). In addition to the alpha abnormalities, abnormal synchronization of theta and beta oscillations was suggested to reflect unstable states of cognitive processing, specifically of working memory in individuals with depression (122). Analysing EEG band power beyond the alpha frequency range provided evidence which suggests that decreased theta power might be important during rumination (42), and lower power in the theta range, as well as alpha frequency band has been noted during mind wandering (43). Moreover, increases in the delta band are generally related to pathology such as mental slowing in dementia (123), as well as psychopathology (124).

### 4.3. Limitations

It was recently shown that machine learning performance in neuroimaging studies of depression overestimate the classification accuracy in small sample sizes (125). This is a well-known phenomenon when the number of features describing the samples exceeds the size of the sample and is not limited to neuroimaging but any modality where the feature vector is long. Certainly, our sample size is very small, as well, especially for the prediction or moderate depressive symptoms in winter. Therefore, we have chosen lasso regularization as an approach to address those problems of high-dimensional feature spaces. The prediction of moderate depressive symptoms might lead to better results because the distinction of the sample is clearer, but it might also be related to the small sample size. Therefore, we chose to not interpret those results any further. However, we also have to question whether the sample composition is representative as women outnumbered men. Although SAD is also more common among women, we had an even higher proportion of women participating in the study, which limits generalizability of the results to men. Future studies need to recruit a significant proportion of men in order to allow for interpretation of gender-specific results.

Another restriction of the study is that it was performed in Iceland, where lighting conditions might not be representative for regions with a lower latitude.

We also need to re-emphasize that this study included the GSS score as a measure for seasonality and other self-assessment questionnaires to measure depressive symptoms, while a clinical interview was not part of the study to ascertain diagnosis of SAD. Therefore, we limit our conclusions to results from a non-clinical sample with mild or moderate depressive symptoms in winter.

### 4.4. Future Directions

The use of psychological characteristics to predict seasonal affective occurrence can be extended to prediction of treatment response. Negative attributional style predicted poor response to pharmacotherapy in nonseasonal depression but not in seasonal affective disorder (19). It was also reported that psychic anxiety was related to response to light therapy while somatic anxiety was rather related to a negative outcome (126), and that atypical symptoms of depression predict responsiveness to light therapy (127). There have also been attempts to predict treatment outcome in order to determine which patients might respond to light therapy (128), or which patients respond better to light therapy, cognitive-behavioral therapy (129), or a combination of the two (130). When patients exhibit cognitive vulnerabilities, the use of cognitive behavior therapy might be crucial (130). In a later study cognitive vulnerability could not be replicated as prognostic or prescriptive predictor of outcome of light- vs. cognitive behavior therapy, but greater morningness was associated with less severe post-treatment depression in both treatment approaches (129). It is possible that EEG-biomarkers could add to the planning of personalized treatment of patients with SAD, both by helping to select the most appropriate therapy alongside with the consideration of cognitive vulnerabilities, as well as by identifying individuals at risk in order to initiate preventative treatment in a timely manner.

## 5. Conclusions

Depressive symptoms in winter may be predicted by self-report questionnaire data better than by EEG measures collected in summer, but the combination of features from both domains is advantageous and leads to higher prediction accuracy.

Our findings on relevant EEG biomarkers emphasize the importance of frontal brain regions in the vulnerability for depressive symptoms in winter as well as a broad frequency range.

## Data Availability Statement

The raw data supporting the conclusions of this article will be made available by the authors, without undue reservation.

## Ethics Statement

We obtained prior approval from the Icelandic National Bioethics Committee on May 28th 2019 (study number 19-090-V1). All investigators signed a non-disclosure contract and written informed consent was obtained prior to inclusion from all participants. The patients/participants provided their written informed consent to participate in this study.

## Author Contributions

YH and RÓ: conceptualization. YH (for EEG): methodology. RÓ (for psychological tests): methodology. YH: software, validation, formal analysis, investigation, data curation, visualization, project administration, and funding acquisition. YH, GK, RÓ, and MU: writing—original draft preparation and writing—review and editing. YH and GK: supervision. All authors have read and agreed to the published version of the manuscript.

## Funding

The study was supported by the Research Fund of the University of Akureyri (RHA, R1916).

## Conflict of Interest

The authors declare that the research was conducted in the absence of any commercial or financial relationships that could be construed as a potential conflict of interest.

## Publisher's Note

All claims expressed in this article are solely those of the authors and do not necessarily represent those of their affiliated organizations, or those of the publisher, the editors and the reviewers. Any product that may be evaluated in this article, or claim that may be made by its manufacturer, is not guaranteed or endorsed by the publisher.
